# On professional skill in the age of digital technology: What happens when work is instrumented with technology

**DOI:** 10.1007/s00146-023-01668-3

**Published:** 2023-04-22

**Authors:** Anders Sandblad

**Affiliations:** Combitech AB, 351 80 Växjö, Sweden

**Keywords:** Professional skill, Tacit knowledge, Tacit engagement, Digitalization, Technology, Turing’s man, Functional autism, The mechanized man

## Abstract

This article is about professional skill and what happens when work is instrumented with technology. The purpose is to contribute to the understanding of the professional skill, its role and development in an increasingly digitalized working life. The article also argues that more research is needed to understand what is at stake in terms of professional skill in the age of digital technology. The research on which the article is based shows that people adapt their way of thinking and perceiving reality to the technology they use. This means that people are gradually becoming more and more like machines. There is an ongoing intellectual inner mechanization, which can be contrasted with the outer mechanization of human muscle power that the industrial revolution entailed. The intellectually mechanized man observes and describes reality in the terms of technology and loses the ability to discern nuances and make qualified judgments gradually. The concepts of Turing’s man and functional autism capture these phenomena. Tacit engagement is a concept that captures the tacit knowledge that can only be expressed when people share physical space. The concept draws attention to the importance of the physical space and the body and what is at stake in terms of interpersonal knowledge in the wake of digital communication technology. It is not machines with supposedly human abilities and characteristics that we need to pay attention to when working life becomes increasingly digitalized, but people who gradually become like machines. What is required to safeguard the knowledge that is unique to man is bildung, i.e., to see the limits of the technology and the abstract theoretical models one uses. Art, classical literature, and drama, with their more plastic language, can reach areas where mathematics and natural science cannot reach.

## Introduction

Digital technology is at the heart of developments in contemporary society and working life. A perspective that has been overshadowed both in research and in the public discourse refers to the nature and development of human professional skill. Instrumentation of work with technology raises several questions of basic epistemological nature: Is it possible to create a machine that can capture all aspects of human knowledge? Is there knowledge that is unique to man? If so, *what happens when tasks are completely or partially transferred to the technology?*

This article draws attention to the last question, more specifically to what happens to the knowledge of the working man when digital technology is used. *The main purpose is to contribute to the understanding of professional skill, its role and development in an increasingly digitalized working life*.

*Another purpose is to create a foundation and encourage further research on professional skill*. For the ongoing digitalization of working life to be sustainable in the long term, brilliant technology, and knowledge of how it works and is designed is not enough. An understanding of the human professional skill and the skill-related limits of technology, as well as insights into what is at stake when the technology is used, is also required.

This article is based on the findings of selected case studies on professional skill from 1970’s onwards and on two recent case studies undertaken by the author on (i) professional skill in the making where the informants consist of young engineers who are new to the professional life, and (ii) on a professional skill during ongoing career, where the informants consist of experienced professionals with different professional backgrounds. These latter two studies applied the *Dialogue Seminar Method* (c.f. Göranzon and Hammarén 2006), with the help of which examples, stories, and reflections on professional skill have been collected. Based on these, a further reflective hermeneutic analysis has been made (see Sandblad 2021).

The findings from this research provide insights regarding the long-term skill-related consequences of the use of technology, and the analysis revives the earlier ground-breaking case-study research on professional skill and computer use that was conducted at the Swedish Centre for Working Life during the 1970s and 1980s. The ideas about the information society at that time have become relevant again today, perhaps more relevant than ever, but now expressed in terms of digitalization and often with AI as an alibi (Lundh Snis et al 2020).

## Professional skill

Professional skill refers to “knowledge that is used in practical situations, to solve tasks you are faced with” (Göranzon 2009, p. 7); knowledge that is expressed in the encounter with individual unique situations and events; the ability to perform judgmental situated *actions* and interventions; knowledge that is not only a fruit of theoretical studies, but also of *practical experience*.

Practical work, regardless of profession, includes various forms of judgements and interventions; a readiness and ability to respond to what the concrete situation requires, even when the circumstances are uncertain. Every situation is unique, which requires attention and discernment. It is when we are faced with something ambiguous, unknown, or unexpected that the knowledge is put to test. It requires the presence of the senses and an ability to both feel and think. The ability to handle the uncertain, the ambiguous, the temporary, the unique and the unexpected is central to skilled, purposeful, and *responsible action* and characteristic of human experience in general (c.f. Janik 1991; Perby 1995; Bergendal 2003).

The ability to handle the ambiguous and the temporary reality distinguishes the knowledge used in practical situations from the formalized theoretical knowledge with its claims of unambiguity and generality, i.e., to be valid everywhere and always (Bergendal 2003, p. 9). Exploring professional knowledge is about paying attention to and examining the tension between the knowledge forms of theory and practice, which cannot be reduced to each other. The two forms of knowledge can be seen as opposites but are at the same time also interdependent. They provide different inputs to the one and same knowledge and qualify each other mutually. The borderland between them is not fixed but in constant motion back and forth (Bergendal 2003, p. 96).

The core of professional skill consists of *tacit knowledge* (Göranzon 1983). This means that there are central aspects in all knowledge that for logical reasons cannot be expressed unambiguously and exhaustively as statements in a natural language or in the form of information and explicit rules (Johannessen 1999, p. 20). Within teams and professions, linguistic communities of action develop where central concepts are understood through pattern-forming actions and examples, rather than through definitions and explicit rules. Wittgenstein (1981, 1992, 2012) calls these communities of action *practice* and a practice has in principle no rules (Johannessen 2007, p. 74). Within a practice, one understands concepts and follow rules in the same way. The understanding of concepts develops through *language games* where different perspectives and experiences meet and fertilise each other. The distinction between following a rule and the rule itself is central. *Rule following* is related to *judgment* and based on *experience*. Ideas to capture all human knowledge in explicit and exhaustive rules are based on a false notion of the perfection of language *the dream of the exact language* (Göranzon 2009, p. 59–82).

At the center of tacit knowledge, we find abilities such as attention, discernment, intuition, and judgment, which are based on a familiarity with the context and the task, acquired through reflected experience. Becoming experienced is not about having been through a lot, but rather about how you have understood what you have been through. By reflecting on your own and others’ experiences in the light of literature and philosophy, you can develop and deepen your understanding (c.f. Hammarén 1999; Fock 2004; Backlund 2006; Ratkic 2006; Sjunnesson 2007; Göranzon 2011).

Both researching and teaching professional skill is about examining and creating a better understanding of the critical core of practical knowledge, its nature and its development. The meaning of examining professional skill can be summarized in terms of expertise, i.e., to better understand what makes a man an expert in his profession; how this expertise is developed and expressed; or what prevents it from being developed and expressed.

## Skill and technology

A connection arises between professional skill on the one hand and technology on the other when the latter is used in work. A skilled professional must of course master his tools, but the tension between the complexity of practice and the rationality of technology has a deeper impact on the relationship between humans and technology, which can be difficult to discern without insights into the nature of professional skill. Computers and other digital technologies are based on storage, transmission, and processing of information, but human expertise is significantly more complex than that.

Aristotle (2012, pp. 161–185), for example, divides knowledge into three forms: *episteme*, *techne* and *fronesis*. Episteme is equal to information and facts – *knowing that*; techne is the knowledge of doing – *knowing how*; and fronesis is the knowledge of judgment – *knowing when*. When knowledge of the forms of techne and fronesis are reduced to information and information processing, losses may arise that jeopardise the outcome and quality of work.

### Eras of research

**Fig. 1:**
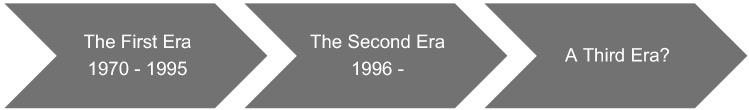
Eras in the research on professional skill

Contemporary research on professional skills has its roots in case studies from the 1970s and 1980s. The case studies were a reaction to the ideas of the time about AI and the information society. The research area has been developed gradually, first at the Swedish Centre for Working Life and later at the Royal Institute of Technology in Stockholm. The development can be roughly divided into two eras. The sections below briefly describe each era and argue for the need for further research – a third era (Fig. 1).

*The age of digital technology*, which society and working life today are entering or already have entered, calls for a need, not only to pay attention to the results of previous case studies but also for new case studies on professional skill and the use of technology.

### The first era

Technological development is often associated with progress. When new and advanced technology is introduced, there are often notions that work will be better performed and / or easier to perform.

During the 1970s and 1980s, there were notions and ideas about the great potential of computers and the possibility of streamlining several different operations. The ideas were similar to today’s ideas about digitalization. A development was predicted where human expertise, not least decision-making, would be transferred to computers. It was considered important to quickly educate people in the new technology and introduce a computer mind, i.e., an ability of professionals to think and work in accordance with the computer systems that were being introduced (Göranzon 2009, p. 131).

As a reaction to these ideas, a series of groundbreaking case studies were conducted at the Swedish Centre for Working Life. The researchers were interested in the long-term skill-related consequences of computerization. The case studies examined, among other things, how foresters’ knowledge of valuing forest properties was affected when the calculations were transferred to computers (Göranzon 2009, pp. 29–57); how the knowledge of administrators at the Social Insurance Offices in Sweden was affected when part of the regulations for sickness payments were computerized (Göranzon 1983, 2009; Josefson 1985, p. 101–110); and how meteorologists’ knowledge of making short-term local weather forecasts was affected when computer-generated weather maps were introduced as an aid (Perby 1988a, b, 1990).

Mentioned case studies form the core of *the first era of research on professional skill*. Consistent themes in the studies were the phenomenon of tacit knowledge, differences between different professional languages and the conditions of understanding, as well as the conditions for certainty in action. One thing the researchers noticed was that the ability to make qualified and reliable judgements was eroded after about 4–5 years of computer use (Göranzon 1983, p. 12–13).

In a Japanese futures study in 1985, the concept *functional autism* (Göranzon 2009, p. 132) was used as a term for how people who had used computers for a long period of time lost their ability to discern the nuances of reality, handle confrontations between different perspectives and make qualified judgements. The computers’ demands for categories and unambiguity had affected their perception of reality and hence eroded their professional skill.

A related concept ascribed to David Bolter (1984) is *Turing’s man*. Bolter argued that Alan Turing’s idea of a machine that can imitate human behavior perfectly (Turing 1950), creates a false image of man as an information processor and reality as information to be processed. Turing’s man as a phenomenon means that man, after close and prolonged use of computers, begins to describe reality in terms of the conceptual world of computers. When technology defines language, it also affects people’s perception of reality and thinking, which has long-term consequences for their judgment.

In an environment of computers and information technology, we also begin to think about humans in mechanical terms. “By making a machine think as a man, man recreates himself, defines himself as a machine”, wrote Bolter (1984, p. 13). Hence, a consequence of the spread of digital technology is that humans have increasingly difficult to understand what separates them from machines.

The skill-related phenomena that the researchers were confronted with could not be interpreted within the common framework of epistemological theories (Hammarén 1999, p. 24). Alternative perspectives and methods were therefore sought, and a new paradigm for working life-oriented research on the use of technology emerged. The paradigm is characterised by its way of illuminating paradoxes and dilemmas that manifest themselves in the field of tension between codified model-based knowledge and experience-based tacit knowledge (Janik 2009).

Theoretically and methodologically, the paradigm is based on an epistemology consisting of four perspectives for reflection: (i) philosophical texts of ideas and science; (ii) Wittgenstein’s philosophy; (iii) art, classical literature, and drama; and (iv) case studies, stories, and examples of professional skill (Göranzon 2009). The purpose is to make human experience-based tacit knowledge accessible for reflection. The knowledge can thus be made visible and qualified.

*Art as a source of knowledge* is what distinguishes research on a professional skill from other working life-oriented research. Due to its more plastic language, art, literature, and drama can make visible an internal perspective on technology relative to the people using it (Göranzon et al 1978; Göranzon 2001).

### The second era

In 1995, *skill and technology* was established as a research area at the Royal Institute of Technology in Stockholm. It was the start of *the second era of research on professional skill*. The case studies conducted did not have the same relation to computer use but rather dealt with different aspects of human professional knowledge and how it develops, professional knowledge within different professional groups, and instrumentation of work with formal processes, instructions, and metrics in relation to experience-based knowledge. The concept of technology thus gained an expanded meaning that not only included technical systems and tools but also the formalization of work and learning in general. Several case studies dealt with various skill-related aspects of engineering and leadership, but studies related to the skill of professional categories such as teachers, nurses and operating staff in the nuclear power industry were also included in the second era.

The most important contribution during the second era of research was a deeper understanding of conditions and methods for exploring and developing the experience-based knowledge of both individuals and collectives. During the first era, methods began to take shape based on the above-mentioned four perspectives for reflection. During the second era, these methods were given structure through a formalization of the *Dialogue Seminar Method* (c.f. Hammarén 1999; Fock 2004; Backlund 2006). Today, it is an established and well-proven method for both exploring and developing professional skill. The method has an ability to call forth examples and stories about personal experiences and stimulate reflection. In a series of seminars with a group of professionals, it is possible to gradually approach their professional skill and allow indirect descriptions and interpretations to grow over time (Göranzon 2001; Backlund 2006; Ratkic 2006).

The three main elements of the method are: (i) individual reflection on personal experiences when reading selected texts of an epistemological nature; (ii) continued individual reflection when writing an essay on personal experiences; and (iii) dialogue-based reflection in group, based on the participants’ personal essays. The dialogue is moderated by a seminar leader and documented in minutes that capture the main thoughts and ideas that emerged from the dialogue. The minutes are returned to the group to be used as a source for continued reflection and dialogue (c.f. Göranzon and Hammarén 2006).

## The mechanized man

In the wake of the technological developments over the past decade, one can see how the amount of administrative computer systems has increased in many operations. An example from the thesis *Professional-skill Machines Humans* (Sandblad 2021) illustrates the consequences of this. A manager with many years of experience from Swedish industry reflected on his experiences:

I am far too often struck by how much time I spend satisfying the needs of different tools. Tools that are also often completely inflexible regarding what information is to be entered, in what place and in what form. I imagine that I do a better job the more tool-like I become. I am suddenly struck by the fact that I walk around thinking about how similar, or at best different from, the tools my colleagues are. Could it be that the more we work with our […] systems, the more similar we become to them?

What the managers reflection shows is that systems that are meant to help rather may take professionals as hostage. They are forced to adapt to the systems and are captured by the mainly administrative task of providing them with the information they require. The systems thus divert attention from the core of the work.

### Technology as tool or system?

The example also shows the metaphorical difference between considering technology in terms of *tools* that function as man’s extended arm, or *systems* where man is no more than a cog in larger machinery. In the latter case, it is more about the computer governing rather than supporting the professional in his work.

The relationship between a tool and its users can be understood in the light of an anecdote about the famous author Ernest Hemingway when he met a photographer at a photo exhibition:

Hemingway asked a photographer “It was a very good picture, what camera did you use” and got the answer “I liked your book – The old and the sea – what typewriter did you use for it?” (Göranzon and Mouwitz 2005, p. 114)

The anecdote of the meeting has two points in this context. The first is that the critical knowledge does not reside in technology but in man. A good typewriter, for example, does not formulate the text for the author. A skilled professional must, of course, master his tools, but that is not enough. The critical knowledge of an expert is more complex than that.

In connection with this, one can also reflect on what man would be without the technology and tools he has created. A pen, a typewriter, and a word processor, for example, provide different conditions and opportunities for writing a book and the result is probably affected by it. In the same way, different cameras provide different conditions and opportunities to take a good photo. An amateur can, for example, take decent photos with the camera in his mobile phone, but does not master a professional camera very well.

Conversely, a professional photographer would be limited if he only had a mobile phone to take photos with; he needs greater degrees of freedom and flexibility than the phone can offer. The photographer Peter Gullers wrote that an automatic camera.

[…] excludes me from my memories and blunts my perception and ability to see nuances. The tacit familiarity is not linked to what I do when I take a picture, i.e., which actions I perform, but to concrete experiences at the time of photography and in the production of pictures. (Gullers 1983, p. 34)

In a metaphorical sense, the automatic camera also transforms the photographer into a machine by limiting his scope for action. This leads to the second point of the Hemingway anecdote: there must be a space for professional skill between the tool and the user, a space for the user to actively use his senses, his familiarity, and his judgment – his experience. The automatic camera passivates the photographer in a way that makes him lose his ability in the long run.

### Man becoming a machine

The manager’s reflection above also contains an aspect of power. Administrative systems are often introduced to relieve someone in an organisation. In the current example, it was a matter of simplifying the work for certain staff at the company. At the same time, it gave them an increased control and thus power over their respective areas. On the other hand, however, the consequence was an increased administrative workload for managers, and communication about and with people was carried out through the systems to a greater extent, rather than in direct contact with the people involved.

Centrally implemented administrative systems can have a governing effect by forcing employees in an organisation to use them for the organisation’s processes to work. It may then become more important to work in accordance with the requirements and conditions of the systems than to do a good job in the current specific situation. What has happened then is a diversion of professional responsibility away from the matter, away from the core of the work and what the task basically is about.

When the systems dictate the conditions, they become governing rather than a support for work. “We serve the machines that were actually intended to serve us,” Weizenbaum and Wendt (2015, p. 148) write. “The relationship has turned around: We have become the servants.”

Sällström (1999) argues in the same spirit that human intelligence is inevitably shaped by the technology that express and maintain human culture:

In our culture, intelligence is shaped by education, conventional coexistence, and constant attending of all the things and tools that are an expression of and maintain this culture. The intelligence of a civilised person is to a large extent already ‘artificial’. Automation in working life is prepared by increasingly designing tasks to suit this artificial element of intelligence. The machine, the computer, can appear human because man has been forced to appear as a machine. (Sällström 1999, p. 116)

In the age of digital technology, culture is shaped by technology and so is human intelligence. When the capacity of a machine on a general level is perceived as superior to human capacity, it does not necessarily depend on the machine as such, but rather on the fact that humans have been reduced to machines. These are gradual changes that take time and can be difficult to perceive.

In summary, what the above shows is that humans meet the machines on the terms of the machines. Humans adapt their way of thinking, perceiving, and describing reality and thus becomes more and more like a machine on an intellectual level – *a mechanized man*. *Functional autism* and *Turing’s man* are concepts from previous research that also point in this direction. This is an overlooked risk we face as digital technology spread and penetrate more and more areas.

The mechanized man as a phenomenon applies not only to the use of computers and other information and communication technology but to technology in the broadest sense. It may, for example, also apply to how tasks and activities are formalized with processes, routines, checklists, and metrics.

Both Sjunnesson (2007) and Berglund (2013) show how distinctly process- and routine-based ways of leading operations risk diverting attention and responsibility away from the matter and the task at hand, towards the processes and routines. For example, when someone reacts to a failure with the attitude that “we only followed the routines” or “we only did what the system told us”, it means that they have taken responsibility for following the system or the routines, but not for the actual task and its outcome. “Can I stand for this?” is a question that has not been addressed properly. This phenomenon marks a shift away from the judgmental towards the formalized; a shift away from rule-following towards the rules themselves, to express it in terms of Wittgenstein’s philosophy.

## Tacit engagement

Another example from the thesis *Professional-skill Machines Humans* (Sandblad 2021) sheds light on another aspect of the mechanized man in relation to the use of digital communication technology. Not least during the Covid-19 pandemic, the use of digital solutions for meetings and teleworking have increased significantly. A manager with several years of experience reflected on the consequences of this:

I realise that I’m trapped. […] My senses are shielded. I get to look through the funnel we call [Microsoft] Teams. Or the phone. I miss my colleagues. Something happens when you meet. […] It’s not fun to work so much by myself and I also do a poorer job. I'm impeded. I must find a way to sharpen my senses. A bit like the blind person must sharpen his other senses to compensate for the loss of vision.

“The funnel we call Teams” stands as a metaphor for the screen limiting the senses. Communication technology has made the world smaller by making it easier to communicate and interact with people who are not at the same physical location. The place where you communicate and collaborate has moved to the internet. It has become virtual and is completely independent of physical space and geography. Another view is that the physical space has been dissolved; it has ceased to exist. As a result, bodily presence has also been excluded from interpersonal communication. In this way, technology has rather moved people further apart and made interactions increasingly superficial.

The phenomenon can be understood through Satinder P. Gills’ (2015) concept of *tacit engagement* and her distinction between *transactional and relational communication*. Gill is interested in what is required of the interface between man and technology to mediate interpersonal communication when people are not physically in the same place. Digital technology is often assumed to be able to do this. This notion is based on a view of knowledge and communication that has its origins in cognitivism and rationalism, where knowledge is reduced to information, thought to algorithmic calculation and communication to the transmission of unambiguous messages. Communication and knowledge are then placed outside of ourselves. The situated character of practical knowledge is excluded, as is the knowledgeable and learning human being. Gill consequently distinguishes between *personal and non-personal knowledge*, where the former is embodied in the individual human being and not transferable in a direct sense.

What digital communication technology mediates, even when it includes not only text but also sound and image, is the transfer of knowledge in the form of information. communication consists of *transactions*, i.e., transmission of messages from a sender to a receiver to achieve a specific goal or purpose. However, the mediation that takes place in the interface between two people is significantly more complex. An interface that supports how we interact and relate to other people must include the body and interpersonal meaning-making.

Communication between people is not only transactional but also has a relational dimension. Technology that fully supports interpersonal communication must mediate both. *Relational communication* includes not only the verbal language, but also how we understand each other and act together through actions, bodily movements, gestures, and faces. These are indirect and sometimes subtle, but at the same time completely indispensable elements in interpersonal communication. They help us dealing with ambiguities, solve problems, identify differences, make judgments, feel moods, cooperate, etc.

Relational communication contains elements of ethics, aesthetics, and empathy. It is based on tacit knowledge and includes not only logical reason but also the body and the senses. The body, place and time are central elements. When we share physical space with others, we can perceive things that technology cannot convey, and we can act and react with a different timing. What happens when technology is introduced as a mediating intermediary for human communication is that parts of the relational dimension risk being lost. The difference is obvious, for example, between sending someone an email or meeting face to face. Neither a phone call nor a video conference can fully replace a physical meeting.

Gill states that our bodies’ movements and positions in space in relation to each other affect how we understand, cooperate, and solve problems together. It is a relational and often overlooked part of interpersonal communication that cannot be reduced to transactions of information. For example, when people collaborate in creative meetings in front of a whiteboard, physical interventions take place that convey a form of tacit knowledge. Gill calls this phenomenon *tacit engagement*.

Transactional communication is based on sameness and agreement. Its goal is consensus, and its premise is that the sender and receiver understand the message or object of knowledge that is communicated in the same way. With such an approach, any misunderstandings have to do with the transmission itself, errors that occur on the communication channel. One thing that is then missed, apart from the importance of the body and place, is that people’s difficulties in understanding each other can sometimes have to do with hearing problems, speech difficulties, language skills or difficult words, but that there is a more basic epistemological problem in that we have different experiences and thus have different notions of reality (Josefson 1985, p. 12).

Relational communication, unlike transactional communication, is as much about disagreement as it is about agreement, according to Gill (2015). Conducting a genuine dialogue is a form of relational communication. It is as much about listening as it is about speaking. Silence can sometimes be more telling than the speech itself. Dialogue also includes more than the words that are said or not said. Physical expressions and both physical and mental presence must also be included. However, it is not just a matter of transmitting gestures and faces that correspond to, reinforce, or nuance the corresponding verbal language expressions. This notion is rather an expression of a transactional view of communication. The opportunity for relational communication and tacit engagement is reduced when we, for example, talk on the phone or have video meetings, in comparison with meetings where the presence is physical, in the same physical room.

During the 1970s and 1980s, the phenomenon of tacit knowledge created opportunities to better understand what is lost if one considers language as the bearer of all knowledge. Tacit engagement today creates similar opportunities to better understand what is lost when people do not share physical space. When the relational dimension of a meeting between people is reduced to transactions, it risks creating a form of functional autism that has to do with interpersonal relations and knowledge of human nature. Sherry Turkle (2017) states, for example, that children who use social media extensively, impair their empathic ability and are less able to have long and deep personal conversations. There are reasons to be aware of what happens in the long run with these interpersonal abilities as physical encounters are increasingly replaced by virtual ones.

## Bildung and technology

Undoubtedly, man have historically benefited greatly from technology. During the industrial revolution, technological development contributed to the mechanization of human bodywork – an *outer mechanization*. Man succeeded in creating machines that could complement, replace, and surpass human muscle power, with an accelerating development of the social and economic prosperity of the industrialised part of the world as a result.

In the wake of digital technology, a development at least as significant and revolutionary as during the industrial revolution is often predicted. The idea is that digital technology should be able to complement, replace and surpass human intelligence, intellectual power, and creative ability in a similar way – an *inner mechanization*.

The meaning of *the mechanized man* as a phenomenon is that the digital technology creates an intellectual inner mechanization of the man himself. When the use of technology in various forms is pushed too far, something risks being lost – operations become *over-digitalized* or *over-formalized*. Thus, there is a skill-related limit where the disadvantages of technology and formalization of work outweigh the advantages, a turning point where the structure, models and rules are transformed into a negative factor. This limit is more important to pay attention to than the idea of a machine that is supposed to have human capabilities and be able to replace or surpass humans.

A way to understand this limit is through Rene Descartes’ division of man into body and soul. Descartes is usually regarded as one of the foremost representatives of the medieval mechanical worldview and of rationalism, i.e., the idea that all knowledge can be achieved through rational thinking. However, he realised that there is a limit to what we can understand and explain with rational mechanistic thinking. The fact that the body—the outer—can be explained in mechanical terms does not mean that the whole human can be explained in this way. What Descartes noticed was that the human soul—the inner—is too complex to be understood rationally. To him the soul was the connection with our divine creator. He thus left the field free for religion (Shapin 2018, p. 159).

Both humans and human life is too large and complex to be reduced to information and calculation or to be given any other simple and unambiguous explanation. The perspectives of mathematics and natural science are not sufficient. Descartes realised this. So did AI pioneer Joseph Weizenbaum who in the same spirit wrote that “real life is not computable” (1980, p. 114; Göranzon 2019, p. 193).

Descartes and Weizenbaum draw a limit for what it is reasonable and appropriate to use the models and methods of mathematics and natural science for. The same limit thus also applies to technology. The ability to see this limit is important; it is to have *bildung* (Göranzon 2011, p. 9).

Mathematics, natural science, and technology can provide us with explanations and solutions to many of the complex phenomena of nature and society but do not reach the complexity and ambiguity that characterises man himself and the reality he lives in. To be able to see and meet this complexity and ambiguity is a way of approaching the concept of bildung. It is a form of knowledge that extends beyond one’s own special field, which requires an overview and includes not only art and literature, but also mathematics, natural science and technology, and a lot more. It is, of course, impossible to master everything, but to realise that things can be different, to maintain different perspectives in parallel and see their limits, is to have bildung.

Bildung is a response to what is required to safeguard and develop the knowledge that is unique to humans in a working life that is becoming increasingly digitalized. Bildung can strengthen the ability to meet the complexity and diversity of life, as well as the ability to think critically and see the skill-related limits of using technology.

Understanding the limits of technology requires an internal perspective relative to the people who have a work relationship with it, and critical thinking about which tasks are reasonable and appropriate to transfer to a machine. Through its more plastic language, art, classical literature, and drama can help us to see and better understand “the long-term aspects of the human–machine relationship through analogous thinking” (Göranzon 2001, p. 12).

By using art, classical literature, and drama as a source of knowledge, it is possible to reach areas that mathematics and natural science can never reach. It can help us to make visible different aspects of human life in all its complexity. It can help us reflect and better understand human dilemmas and paradoxes without predefined or simple solutions; phenomena and “events that we in all their multiplicity and unique character cannot control” (Josefson 1998, p. 77).

## Call for research

If the mechanized man’s way of thinking and perceiving reality spreads in working life and is reproduced in young individuals in school, we risk having a society drained of humanism and judgment, populated by people without access to personal experience-based knowledge; a society characterised by functional autism, where knowledge has been reduced and solidified into information and rules.

An ambition of this article is to create a platform for continued research with a focus on professional skill, called for by contemporary ideas about AI and digitalization—a third era of research. More knowledge is needed to shed light on and draw attention to what the age of digital technology means for the working professional and what is at stake in terms of professional knowledge when operations are instrumented with new technological applications. The risk of *over-digitalization* or *over-formalization*, with unexpected and unwelcome setbacks, is otherwise imminent.

### Tacit engagement

An example of an area to explore further is the consequences of digital communication technology and the increasing use of digital solutions for meetings and teleworking. The concepts of *transactional and relational communication*, as well as *tacit engagement* as a further development of tacit knowledge, can be used as a vehicle for a third era of research to gain insights and better understand the importance of the body and the physical space for professional skill.

### Rethinking tacit knowledge

Another proposal for further development of the phenomenon of tacit knowledge departures from the fact that man in an environment of digital technology tends to think and describe himself in terms of the machine, i.e., that he is having increasing difficulty to understand what separates a human being from a machine.

With the concept *the unsayable*, Wittgenstein (1992, p. 25) adds an existential dimension to tacit knowledge by pointing to what shapes a person and his conception of reality. The unsayable is based on personal experience gathered through life and provides the background against which what can be said has its explanation. It is a form of knowledge that extends beyond the ability to, for example, sense the taste of a strawberry and distinguish it from the taste of a raspberry. Rather, it is about how we encounter, deal with and are shaped by what cannot be foreseen, for example when we are faced with human crises of an existential nature. In this field, mathematics, natural science, and other calculating sciences cannot help us. What we must turn to is instead art, classical literature, and drama.

## Data Availability

The datasets generated during and/or analyzed during the current study are available from the corresponding author on reasonable request.
